# Perceived behavioural predictors of late initiation to HIV/AIDS care in Gurage zone public health facilities: a cohort study using health belief model

**DOI:** 10.1186/s13104-018-3408-4

**Published:** 2018-05-22

**Authors:** Teklemichael Gebru, Kifle Lentiro, Abdulewhab Jemal

**Affiliations:** 1 Department of Public Health, College of Medicine and Health Science, Wolkite University, Wolkite, Ethiopia; 2 Department of Medicine, College of Medicine and Health Science, Wolkite University, Wolkite, Ethiopia

**Keywords:** Perceived behaviour, Late initiation, HIV/AIDS care

## Abstract

**Objective:**

The study was aimed to measure incidence density rate and identify perceived behavioural believes of late initiation to HIV/AIDS care in Gurage zone public health facilities from September 2015 to November 2016.

**Results:**

The incidence density rates of late initiation to HIV/AIDS care were 2.21 per 100 person-months of observation. HIV positive individuals who did not perceived susceptibility were 8.46 times more likely delay to start HIV/AIDS care than their counter parts [OR = 8.46 (95% CI 3.92, 18.26)]. HIV infected individuals who did not perceived severity of delayed ART initiation were 6.13 time more likely to delay than HIV infected individuals who perceived its severity [OR = 6.13 (95% CI 2.95, 12.73)]. HIV positive individuals who didn’t have self-efficacy were 2.35 times more likely delay to start HIV/AIDS care than HIV positive individuals who have self-efficacy [OR = 2.35 (95% CI 1.09, 5.05)].

**Conclusions:**

The study revealed that high incidence density rates of delayed initiation for HIV care and variations were explained by poor wealth, and perceived threat and benefit. Therefore, interventions should be designed to initiate care at their diagnosis time.

**Electronic supplementary material:**

The online version of this article (10.1186/s13104-018-3408-4) contains supplementary material, which is available to authorized users.

## Introduction

### Background

Human immunodeficiency virus (HIV) is a global challenge that affects about 35.3 million people lives with HIV and 1.6 million people kills in 2012 [1, 2]. Sub-Saharan Africa remnants the most seriously affected region by the disease, nearly one in every 20 (4.9%) individuals were infected, this shares 69% of the world population who have HIV infection [3]. Since the index case was detected in Ethiopia in 1984, about 1.3 million individuals living with the virus, and HIV/AIDS killed millions people and lefts 744,100 orphan children [2]. The health status of HIV positive individuals at the time of Antiretroviral Therapy (ART) initiation contributes vital role for their health conditions [3–6].

World Health Organization (WHO) recommends to all HIV infected individuals whose CD4 counts of ≤ 500 cells/µL should start ART regardless of the presence or absence of clinical symptoms and/or individuals with advanced clinical disease (clinical stage 3 or 4) should start ART irrespective of their CD4 cell count threshold, which is currently accepted and applied in Ethiopian [7]. ART starting at CD4 levels higher than 200 or 250 cells/µL increases the survival time by 20%. Furthermore, initiation of ART has also initiated hope to people living with HIV/AIDS that credit to improve the quality of life [8, 9]. However, delayed ART initiation causes less likely to respond the treatment, more likely to burden on healthcare financing, and have a higher mortality rate [4–6].

Health belief model (HBM) was one of the theories designed to explore preventive healthy behaviour if a person has positive expectation. Thus, perception of an individual can be affected by socio-demographic factors, level of knowledge, threat, available interventions and/or their barriers, and influence of others [10].

### Statement of the problem

Significant quantity (15–43%) of HIV positive individuals living in third world countries were came for care very delay with severe disease [8]. Delayed initiation of ART is associated with higher rates of HIV-related morbidity and mortality [4]. In sub-Saharan Africa, the overall coverage of ART initiation was 48% of those in eligible, emerging evidence showed that up to 59% of people living with HIV were either lost to follow-up or started ART at late stage of the disease [3, 4].

In Ethiopia, the coverage of ART was 53% (336,160 persons) of those eligible for the treatment (CD4 cell counts < 500 cells/µL) [8, 11]. In effect, the country planned to increase ART coverage to reach 95%. Perhaps, studies conducted in different area of the country shows the prevalence of delayed presentation to HIV/AIDS care were 40, 38.8 and 33.1%, [12–15].

Delayed presenting hinders people from obtaining maximum benefit of being screened for tuberculosis and sexually transmitted infections (STI), timely ART initiation, and counselling to prevent further opportunistic infections [6, 16–20]. Considering its low prevalence of HIV/care, improvement of ART treatment is a priority intervention area of the Ethiopian ministry of health.

In this regard, there is no published research report on behavioural aspect of ART initiation. Thus, the purpose of the study was to measure incidence density rate and explore perceived behavioural believes that may predispose to late initiation to HIV care that helps to design effective intervention in Ethiopia and beyond.

## Main text

### Method and subjects

#### Study area and period

The study was performed at Gurage zone that has 1,583,824 populations; of these 744,397 were males and 839,427 females. The population of area get healthcare service from five hospitals and 70 health centres by 1526 health professionals. Moreover, 21 health facilities were providing pre-ART and ART services for 4143 and 2597 people, respectively [21–23]. We follow them and interview at their time of initiation about the predictors of initiation as a case–control approach from September 2015 to November 2016.

#### Study design

Cohort study that has both prospective and retrospective components using health belief model was employed. All peoples living with HIV in Gurage zone were source population; whereas people living with HIV linked pre-ART clinics in the study area during the study period were study population.

#### Eligibility criteria

At the time of study in Ethiopia ART enrolment is expected to be started at CD4 count less than 500 cell/µL or WHO clinical stage III or IV. However, HIV infected peoples diagnosed with CD4 count greater than 500 cell/µL were linked to pre-ART clinic for follow-up not yet started ART. Therefore, among the HIV positive individuals linked to pre-ART individuals initiated ART with WHO clinical stage of III or IV or a CD4 count less than 350 cell/µL were included as CASE and for their counterpart individuals who initiates the treatment with WHO clinical stage of I or II or CD4 count more than or equal to 350 cell/µL were included as CONTROL [24–26]. However, HIV positive peoples who are directly initiated the treatment and children less than 18 years’ age were excluded from the study.

#### Sample size determination and sampling technique

The sample size was calculated using STATCALC program of EPI INFO version 7 statistical packages by assuming; the proportion of adult illiteracy among cases 64% (gave maximum sample size) [11], 95% CI 80% power, case to control ratio of 1:1 to detect an odds ratio of 2.0, and 10% non-response rate. Accordingly, the calculated optimal sample size was 320 (160 cases and 160 controls) and consecutive sampling were used.

#### Data collection and quality assurance

Data was collected by trained diploma and above holder health professionals using Amharic version adapted interviewer administered, pre-coded and pre-tested structured instrument. The tool includes socio-demographic characteristics, wealth index computed from 16 items that ranges 0–16, knowledge (12 items of yes or no response that ranges from 0 to 12) and behavioural perceptions including threat (both susceptibility and severity) (11 Likert scale items that ranges from 11 to 55), benefit (5 items that ranges from 5 to 25), barrier (5 items that ranges from 5 to 25), Self-efficacy (4 items that ranges from 4 to 20) and cue-to-action (9 items of yes or no response that ranges from 0 to 9) (Additional file 1) [27–30]). Furthermore, base line data was collected from their medical record such as time of diagnosis, level of CD4 count and WHO clinical stage.

Data collection instrument was pre-tested on 5% of the actual sample size and 3 days training with mock was given to data collector and supervisors prior the process of data collection. Furthermore, double data entry was made using Epi-data software for validation.

#### Data processing and analysis

Statistical packages such as Epi-data 3.1 for data entry, STATA-12 to calculate incidence density rate and SPSS-20 to compute numerical values and identify significant predictors. Level of knowledge was computed after reversely recoding the response it ranges from 0 to 12 and categorised as “informed” and “uninformed” for the value above the mean and below the mean, respectively. For HBM constructs initially, oppositely phrased items were reversely coded and factor analysis were applied to assess; factor-item correlation matrix at least two variables greater than 0.30, Barlett test of sphericity below 0.001 and Kaiser–Meyer–Olkin (KMO) 0.6 or above with Oblique rotation [10]. Furthermore, parallel analysis was used to estimate eigen value [32] and reliability analysis to compute Cronbach alpha coefficient with 0.70 and above was accepted as internally consistent items. Furthermore, care was taken to deal with missing values by excluding cases pairwise. Lastly all the constructs of HBM were dichotomised as “Yes” and “No” response for the value above the mean and below the mean based on each rage, respectively.

Predictor assessment was made by constructing selective model and only variables with p < 0.25 in the binary regression were further considered to final model [33]. Then, backward stepwise logistic regression method was applied to control the effect of confounding factors [34]. Odds ratio (OR) with 95% confidence interval (CI) were used to estimate the strength of association and its stability. For all statistically significant tests, the cut-off value set is p value less than 0.05.

### Results

#### Socio-demographic characteristics

We planned to participated 320 individuals in the study; however, 317 subjects deployed for the study that makes 99.06% response rate. The mean age of the study participants was 33 with 8 standard deviations. Majority of the study participants were females 189 (59.6%) and single by their marital status 131 (41.3%). Most of the respondents’ highest educational level was none or didn’t get formal education 127 (40.1%) followed by College graduates and above 91 (28.7%). Furthermore, half of the study participants stay with HIV/AIDS at least for 5 years 165 (52.1%) (Table 1).

**Table 1:** Socio-demographic characteristics of study participants for late presentation to HIV/AIDS care in Gurage zone public health facilities, n = 317, Sep. 2015 to Nov. 2016

Variable description	ART initiation condition	
	Case	Control
	Count (%)	Count (%)
Gender		
Male	84 (52.5)	44 (28.0)
Female	76 (47.5)	113 (72.0)
Marital status		
Single	64 (40.0)	67 (42.7)
Married	33 (20.6)	23 (14.6)
Divorce	32 (20.0)	44 (28.0)
Widowed	31 (19.4)	23 (14.6)
Highest education		
None or can’t get formal education	49 (30.6)	78 (49.7)
Elementary	35 (21.9)	8 (5.1)
Secondary	36 (22.5)	20 (12.7)
College graduate and above	40 (25.0)	51 (32.5)
Occupational status		
Unemployed	23 (14.4)	12 (7.6)
Student	7 (4.4)	8 (5.1)
House wife	49 (30.6)	74 (47.1)
Employed	81 (50.6)	63 (40.1)
Wealth index		
Poor	67 (41.9)	35 (22.3)
Medium	40 (25.0)	63 (40.1)
Rich	53 (33.1)	59 (37.6)
Time living with HIV		
Less than 1 year	46 (28.8)	31 (19.7)
1–5 years	51 (31.9)	24 (15.3)
Greater than 5 years	63 (39.4)	102 (65.0)

#### Incidence density rate of HIV/AIDS care

The person time incidence rate from pre-ART to ART initiation of HIV positive individuals were 4.46 per 100 person-months of observation. Furthermore, the incidence density rates of delay to HIV/AIDS care were 2.21 per 100 HIV positive person-months of observation.

#### Behavioural factors related to late HIV/AIDS care

More than half of both case and control study participants had have a knowledge on ART 105 (65.6%) and 121 (77.1%), respectively. Twenty-four (15.0%) of the cases and 91 (58.0%) of the control were perceived severe consequences of delay to start HIV/AIDS care. Around one-fourth of the cases 29 (18.1%) and more than half of the controls 93 (59.2%) were perceived to get benefit from early initiation of HIV/AIDS care (Table 2).

**Table 2:** Behavioural factors related to late initiation for HIV/AIDS care in Gurage zone public health facilities, n = 317, Sep. 2015 to Nov. 2016

Variable	ART initiation condition	
	Case	Control
	Count (%)	Count (%)
Knowledge		
Informed	105 (65.6)	121 (77.1)
Uninformed	55 (34.4)	36 (22.9)
Perceived susceptibility		
Yes	33 (20.6)	129 (82.2)
No	127 (79.4)	28 (17.8)
Perceived severity		
Yes	43 (26.9)	133 (84.7)
No	117 (73.1)	24 (15.3)
Perceived benefit		
Yes	29 (18.1)	93 (59.2)
No	131 (81.9)	64 (40.8)
Perceived barrier		
Yes	58 (36.2)	59 (37.6)
No	102 (63.8)	98 (62.4)
Self-efficacy		
Yes	57 (35.6)	93 (59.2)
No	103 (64.4)	64 (40.8)
Cue to action		
Yes	87 (54.4)	59 (37.6)
No	73 (45.6)	98 (62.4)

#### Independent predictors of late initiation to HIV/AIDS care

Uniformed HIV positive individuals about HIV care were 1.94 times more likely to delay HIV/AIDS care than informed individuals [OR = 1.94 (CI 1.06, 3.56)]. HIV positive individuals who did not perceived susceptibility were 8.46 time more likely delay to start HIV care than their counter parts [OR = 8.46 (95% CI 3.92, 18.26)]. HIV infected individuals who did not perceived severity of delayed ART initiation were 6.13 time more likely to delay than HIV infected individuals who perceived its severity [OR = 6.13 (95% CI 2.95, 12.73)]. HIV positive individuals who didn’t have self-efficacy were 2.35 time more likely delay to start HIV care than HIV positive individuals who have self-efficacy [OR = 2.35 (95% CI 1.09, 5.05)] (Table 3).

**Table 3:** Independent predictors of late initiation to HIV/AIDS care in Gurage zone public health facilities, n = 317, Sep. 2015 to Nov. 2016

Predictor variables	ART initiation condition		OR (95% CI)	
	Case	Control	Crude	Adjusted
	Count (%)	Count (%)		
Knowledge				
Informed	105 (65.6)	121 (77.1)	1	1
Uninformed	55 (34.4)	36 (22.9)	3.74 (2.35, 5.96)	1.94 (1.06, 3.56)
Perceived susceptibility				
Yes	26 (16.2)	106 (67.5)	1	1
No	134 (83.8)	51 (32.5)	17.73 (10.13, 31.04)	8.46 (3.92, 18.26)
Perceived severity				
Yes	24 (15.0)	91 (58.0)	1	1
No	136 (85.0)	66 (42.0)	15.08 (8.63, 26.34)	6.13 (2.95, 12.73)
Perceived benefit				
Yes	29 (18.1)	93 (59.2)	1	1
No	131 (81.9)	64 (40.8)	6.56 (3.93, 10.96)	3.12 (1.53, 6.33)
Self-efficacy				
Yes	57 (35.6)	93 (59.2)	1	1
No	103 (64.4)	64 (40.8)	2.63 (1.67, 4.13)	2.35 (1.09, 5.05)

### Discussion

In this study it was revealed that the incidence density for late HIV care were high, this could be explained by after diagnosis peoples may searful to start the care within short period of time. Knowledge about the care was associated to delay the treatment. HIV positive individuals who perceived susceptibility were significantly associated with late presentation to HIV care which is consistent with the studies done in Wollo [35, 36]. The higher likelihood of individuals did not perceived susceptibility for presenting late to HIV/AIDS care could be explained by fearful to start care after diagnosis. HIV positive individuals who did not perceived the seriousness were significantly associated with late presentation to HIV/AIDS care which is consistent with the studies done in Tigray [OR = 4.3, (95% CI 2.26–8)] [37], and Durban [36]. The higher likelihood of individual not perceived seriousness presenting late to HIV care could be explained by lack of information particularly on early initiation (Additional file 1).

HIV positive individuals who did not perceived benefit from HIV care and individuals who had lost self-efficacy were significantly associated with late presentation to HIV care which is consistent with the studies done in Vietnam, and Wollo [30, 35–38]. The higher likelihood of individuals not perceived benefit from the care presents lately could be explained due to social stigma, lack of knowledge, or doesn’t perceiving medical care have advantageous in case of once HIV positive.

### Conclusions

The study revealed that high incidence density rates of delayed HIV care initiation and variation explained by level of uninformed about the care, perceived threat, and self-efficacy. Therefore, programs should be designed for effective counselling focused on the consequence of late ART initiation, adherence and to start HIV care immediately at their time of diagnosis.

## Limitation of the study

This study lacks triangulation with qualitative findings to address unexpected issues and culture based variation. Furthermore, this research may be subjected to recall bias and social desirability.

## Additional file

**Additional file 1.** Questionnaire.

## Abbreviations

AIDS: acquired immune deficiency syndrome
ART: Antiretroviral Therapy
CI: confidence interval
HIV: human immunodeficiency virus
KMO: Kaiser–Meyer–Olkin
OR: odds ratio
SPSS: Statistical Package for Social Science
STI: sexually transmitted infection
WHO: World Health Organization
µL: micro liter
