# Supplementary material for: Perceived behavioural predictors of late initiation to HIV/AIDS care in Gurage zone public health facilities: a cohort study using health belief model
Source: BMC Res Notes. 2018 May 22;11:336. doi: 10.1186/s13104-018-3408-4 (PMC5964917; doi:10.1186/s13104-018-3408-4)
Supplement: Supplementary file 1 — Additional file 1. Questionnaire. [file 13104_2018_3408_MOESM1_ESM.docx]

**Questionnaire**

**Part I: General information:**

| **No** | **General information** | **Possible response categories** | **Skipping** |
| --- | --- | --- | --- |
| 001 | Health facility Name | _____________________ |  |
| 002 | District Name | _____________________ |  |
| 003 | Length of time living with HIV | < 1 year 1–3 years 3–5 years > 5 years Don’t know |  |
| 004 | Date of arrival at pre-ART | _____ dd ______mm/2008 |  |
| 005 | CD4 count level (at pre-ART arrival) | _____________ cells/mm^3^ |  |
| 006 | Date of interview (ART initiation date) | _____ dd ______mm/2008 |  |
| 007 | CD4 count level (at ART initiated date) | _____________ cells/mm^3^ |  |
| 008 | WHO clinical stage (Review the stage from document.) | 1. Stage I 2.Stage II  3. Stage III 4.Stage IV |  |

**Part 1I: Socio-demographic Factors**

| **No.** | **Item** | **Possible response categories** | **Skipping** |
| --- | --- | --- | --- |
| 100 | Age | _________________ (in year) |  |
| 101 | Gender | 1. Male 2. Female |  |
| 102 | Marital status | 1. Single 2. Married 3. Divorce 4. Widowed |  |
| 103 | Highest Education Level | 1. Can’t Write and read 2. Write and read only 3. Elementary 4. Secondary 5. College graduate 6. University graduate |  |
| 104 | Occupational status | 1. unemployed 2. Student 3. House Wife 4. Employed |  |

**Part III: Wealth Index Related**

| **No** | **Name of the variable** | Response category |
| --- | --- | --- |
| 200 | Material of the house | 1 = concrete or wood  0 = mud |
| 201 | Roof material | 1 = tiles or galvanized iron or concrete  0 = mud or thatch or plastic |
| 202 | Crowding | 1 = 5 or less people per room  0 = 6 or more people per room |
| 203 | Type of lighting | 1 = electricity or gas  0 = candle or wood |
| 204 | Source of water | 1 = piped into dwelling or borehole with pump or protected dug well  0 = pond or unprotected well |
| 205 | Toilet facilities | 1 = flush or ventilated improved latrine  0 = open pit or none (bush field) |
| 206 | Has a radio | 1 = yes  0 = no |
| 207 | Has a TV | 1 = yes  0 = no |
| 208 | Has a stove | 1 = yes  0 = no |
| 209 | Has a fridge | 1 = yes  0 = no |
| 210 | Has a mobile phone | 1 = yes  0 = no |
| 211 | Has a bicycle | 1 = yes  0 = no |
| 212 | Has a motorbike | 1 = yes  0 = no |
| 213 | Has a car | 1 = yes  0 = no |
| 214 | Livestock | 1 = yes  0 = no |
| 215 | Land ownership | 1 = yes  0 = no |

**Part IV: Knowledge**

| **No** | **Variable** | | | **Response** |
| --- | --- | --- | --- | --- |
| 301 | HIV is spread by | Kissing | | 1. Yes 2. No |
|  |  | Sexual intercourse | | 1. Yes 2. No |
|  |  | Mosquitoes | | 1. Yes 2. No |
|  |  | Coughing | | 1. Yes 2. No |
|  |  | Blood transfusion | | 1. Yes 2. No |
|  |  | Infected bodily fluids | | 1. Yes 2. No |
| 302 | CD4 count for ART initiation | | | 1. Less than 500 2. Less than 300 |
| 303 | If you are HIV positive your children will definitely be positive | | | 1. Yes 2. No |
| 304 | Antiretroviral therapy can cure HIV | | | 1. Yes 2. No |
| 305 | Action of antiretroviral therapy | | Kills the virus | 1. Yes 2. No |
|  |  |  | Reduces the viral load in the blood | 1. Yes 2. No |
|  |  |  | Makes it safe to have unsafe sex | 1. Yes 2. No |
| 306 | ART use can increase survival time | | | 1. Yes 2. No |

**Part IV - Health Belief Model domain**

**Perceived susceptibility to late initiate of HIV care**

| **No.** | **Items** | **Possible response categories** |
| --- | --- | --- |
| 400 | I belief I will be ill due to the acquired HIV/DIDS, if I am lately initiated ART drug. | 1. Strongly Disagree 2. Disagree 3. Neither Agree nor Disagree 4. Agree 5. Strongly Agree |
| 401 | PLWHA peoples are at risk for getting opportunistic diseases, if they are lately initiated ART drug. | 1. Strongly Disagree 2. Disagree 3. Neither Agree nor Disagree 4. Agree 5. Strongly Agree |
| 402 | I belief I do get the opportunistic infection due to lately initiated the ART drug. | 1. Strongly Disagree 2. Disagree 3. Neither Agree nor Disagree 4. Agree 5. Strongly Agree |
| 403 | I am early initiated the ART drug and I do not get any opportunistic infections. | 1. Strongly Disagree 2. Disagree 3. Neither Agree nor Disagree 4. Agree 5. Strongly Agree |
| 404 | Failing to know HIV status early would lead to late initiation of ART drug. | 1. Strongly Disagree 2. Disagree 3. Neither Agree nor Disagree 4. Agree 5. Strongly Agree |
| 405 | I belief failing to know CD_4_ status early may lead to late initiation of ART drug. | 1. Strongly Disagree 2. Disagree 3. Neither Agree nor Disagree 4. Agree 5. Strongly Agree |

**Perceived severity of the late initiate of HIV care**

| **No** | **Items** | **Possible response categories** |
| --- | --- | --- |
| 406 | If I get the ART drug lately I will get sick. | 1. Strongly Disagree 2. Disagree 3. Neither Agree nor Disagree 4. Agree 5. Strongly Agree |
| 407 | If I get the ART drug lately I will lose income. | 1. Strongly Disagree 2. Disagree 3. Neither Agree nor Disagree 4. Agree 5. Strongly Agree |
| 408 | Late ART drug will lead to disabilities. | 1. Strongly Disagree 2. Disagree 3. Neither Agree nor Disagree 4. Agree 5. Strongly Agree |
| 409 | If I get the ART drug lately HIV/AIDS is probably becomes the worst disease. | 1. Strongly Disagree 2. Disagree 3. Neither Agree nor Disagree 4. Agree 5. Strongly Agree |
| 410 | I belief lately initiating ART drug will lead to fasten being infected by opportunity disease. | 1. Strongly Disagree 2. Disagree 3. Neither Agree nor Disagree 4. Agree 5. Strongly Agree |

**Perceived HIV care benefit**

| **No.** | **Item** | **Possible response categories** |
| --- | --- | --- |
| 411 | If I early initiated the ART drug, I will not get early sick from the acquired HIV/ADIS. | 1. Strongly Disagree 2. Disagree 3. Neither Agree nor Disagree 4. Agree 5. Strongly Agree |
| 412 | Early ART drug initiation prevents opportunistic infections. | 1. Strongly Disagree 2. Disagree 3. Neither Agree nor Disagree 4. Agree 5. Strongly Agree |
| 413 | Early initiation of ART drug prolongs the life expectancy of PLWHA. | 1. Strongly Disagree 2. Disagree 3. Neither Agree nor Disagree 4. Agree 5. Strongly Agree |
| 414 | Early getting of ART drug helps to get the next health care benefits that improve health. | 1. Strongly Disagree 2. Disagree 3. Neither Agree nor Disagree 4. Agree 5. Strongly Agree |
| 415 | Early initiation of ART is better than lately initiation of ART for one’s health. | 1. Strongly Disagree 2. Disagree 3. Neither Agree nor Disagree 4. Agree 5. Strongly Agree |

**Perceived barriers to late initiate of HIV care**

| **No** | **Items** | **Possible response categories** |
| --- | --- | --- |
| 416 | I will have suffered by side effects from the ART drug. | 1. Strongly Disagree 2. Disagree 3. Neither Agree nor Disagree 4. Agree 5. Strongly Agree |
| 417 | I afraid that the health care providers who offer the treatment will never keep results really confidential. | 1. Strongly Disagree 2. Disagree 3. Neither Agree nor Disagree 4. Agree 5. Strongly Agree |
| 418 | I will get social discrimination. | 1. Strongly Disagree 2. Disagree 3. Neither Agree nor Disagree 4. Agree 5. Strongly Agree |
| 419 | I become shay if somebody see me when I going to or wait at ART clinic. | 1. Strongly Disagree 2. Disagree 3. Neither Agree nor Disagree 4. Agree 5. Strongly Agree |
| 420 | There is payment for the ART drug. | 1. Strongly Disagree 2. Disagree 3. Neither Agree nor Disagree 4. Agree 5. Strongly Agree |

**Self-efficacy**

| **No.** | **Items** | **Possible response categories** |
| --- | --- | --- |
| 421 | I am confident that I will not got opportunistic infection, if I early started ART drug. | 1. Strongly Disagree 2. Disagree 3. Neither Agree nor Disagree 4. Agree 5. Strongly Agree |
| 422 | People may experience fear to use ART service in transparent way after getting tested to be HIV positive, I am confident to transparently use ART service. | 1. Strongly Disagree 2. Disagree 3. Neither Agree nor Disagree 4. Agree 5. Strongly Agree |
| 423 | I can freely continue my daily routine activities as previous in spite of ART drug initiation. | 1. Strongly Disagree 2. Disagree 3. Neither Agree nor Disagree 4. Agree 5. Strongly Agree |
| 424 | I am confident to I have started ART drug when it was needed. | 1. Strongly Disagree 2. Disagree 3. Neither Agree nor Disagree 4. Agree 5. Strongly Agree |

**Cues to late initiate of HIV care**

| **No.** | **Item** | **Possible response categories** | **Confirmation** |
| --- | --- | --- | --- |
| 425 | Sources of Information about ART drug | Media | 1. Yes 2. No |
|  |  | Family member | 1. Yes 2. No |
|  |  | Partner | 1. Yes 2. No |
|  |  | Friend | 1. Yes 2. No |
|  |  | Health professionals | 1. Yes 2. No |
|  |  | ART drug users | 1. Yes 2. No |
| 426 | who would you Recommended to start ART treatment | Physician | 1. Yes 2. No |
|  |  | Pharmacist | 1. Yes 2. No |
|  |  | Nurse | 1. Yes 2. No |
